# Retraction Note: Knockdown of linc-UFC1 suppresses proliferation and induces apoptosis of colorectal cancer

**DOI:** 10.1038/s41419-026-09339-z

**Published:** 2026-10-01

**Authors:** T. Yu, T-D Shan, J-Y Li, C-Z Huang, S-Y Wang, H. Ouyang, X-J Lu, J-H Xu, W. Zhong, Q-K Chen

**Affiliations:** Department of Gastroenterology and Guangdong Provincial Key Laboratory of Malignant Tumor Epigenetics and Gene Regulation, Sun Yat-Sen Memorial Hospital, Sun Yat-Sen University, Guangzhou, Guangdong China

Retraction Note to: *Cell Death & Disease* https://doi.org/10.1038/cddis.2016.124, published online 19 May 2016

The Editors have retracted this article. After publication, concerns were raised regarding similarities between this article and the authors' earlier article [1], specifically:

- Fig. 4a LOVO NC. LOVO sh#2 and SW480 NC plots appear highly similar to Fig. 4a SW480 si#1, LOVO si#2 and SW480 si#2 in [1], respectively;
- Fig. 5a SW480 β-actin lanes 1&2 appear highly similar to Fig. 7b RKO β-actin lanes 2&3 in [1];
- Fig. 6a SW480 p-JNK lanes 1&2 appear highly similar to Fig. 7b LOVO Atg5 lanes 2&3 in [1];
- Fig. 6a SW480 p-ERK1/2 blot appears highly similar to Fig. 7b RKO Atg7 in [1], stretched vertically;
- Fig. 6a LOVO p-ERK1/2 lanes 2&3 appear highly similar to SW480 p-ERK1/2 lanes 1&2, stretched vertically.

The authors have stated that errors occured in data management and storage. The Editors therefore no longer have confidence in the presented data.

The authors have not responded to any correspondence from the editor or publisher about this retraction.
